# Properties of a brief assessment tool for longitudinal measurement of cognition in people living with HIV

**DOI:** 10.1371/journal.pone.0213908

**Published:** 2019-03-25

**Authors:** Marie-Josée Brouillette, Lesley K. Fellows, Lois Finch, Réjean Thomas, Nancy E. Mayo

**Affiliations:** 1 Department of Psychiatry, Faculty of Medicine, McGill University, Montreal, Quebec, Canada; 2 Chronic Viral Illness Service, McGill University Health Center, Montreal, Quebec, Canada; 3 Research Institute of the McGill University Health Centre, Montreal, Quebec, Canada; 4 Department of Neurology and Neurosurgery, Faculty of Medicine, McGill University, Montreal, Quebec, Canada; 5 Montreal Neurological Institute, McGill University, Montreal, Quebec, Canada; 6 Division of Clinical Epidemiology, McGill University Health Center, Montreal, Quebec, Canada; 7 Clinique médicale l’Actuel, Montreal, Quebec, Canada; 8 School of Physical and Occupational Therapy, Faculty of Medicine, McGill University Montreal, Quebec, Canada; Cardiff University, UNITED KINGDOM

## Abstract

**Background:**

Mild cognitive impairment is common in chronic HIV infection and there is concern that it may worsen with age. Distinguishing static impairment from on-going decline is clinically important, but the field lacks well-validated cognitive measures sensitive to decline and feasible for routine clinical use. Measures capable of detecting improvement are also needed to assess interventions. The objective of this study is to estimate the extent of change on repeat administration of three different forms of a brief computerized cognitive assessment battery (B-CAM) developed for assessing cognitive ability in the mildly-impaired to normal range in people living with HIV. We hypothesized no change over a six-month period in people on effective antiretroviral therapy.

**Methods:**

102 HIV+ individuals completed a set of computerized cognitive tasks on three occasions over a six-month period. Rasch analysis was used to determine if change over time (i.e. improvement due to practice) was uniform across tasks and to refine scoring in order to produce three forms of the B-CAM of equivalent level of difficulty. Group-based trajectory analysis (GBTA) was then applied to determine if performance at baseline influenced the magnitude of practice-related improvement on the battery as a whole over the course of follow-up.

**Results:**

Two cognitive tasks (fluency and word recall) had different levels of difficulty across test sessions, related to the different forms of the tasks. These two items were split by testing session. For all other items, the level of difficulty remained constant across all three time points. GBTA showed that the sample was composed of three distinct groups of people with unique trajectories, defined mainly by level of cognitive ability at baseline. Only the highest group showed an apparent improvement over time, but this change fell within measurement error.

**Conclusions:**

Rasch analysis provides mathematical confirmation that these three forms of the B-CAM are of equivalent difficulty. GBTA demonstrates that no adjustment of the total score is required to correct for practice effects. Application of these modern statistical methods paves the way towards rapid and robust quantification of change in cognition.

## Introduction

HIV-Associated Neurocognitive Disorder (HAND) has been reported in as many as 30–60% of people living with HIV [1–4]. Its course is not uniform across individuals [5] and the underlying causes and appropriate treatment remain uncertain in those with full viral suppression. Despite the high prevalence, expected to further increase as more people live longer with HIV, front-line clinicians lack the tools they need to measure cognitive ability and detect decline over time, particularly in patients with mild cognitive impairment. Repeat neuropsychological testing is not available or feasible in most settings where such patients receive their care. Existing freely-available cognitive screening tests do not have the sensitivity required to detect the milder cognitive impairment that is the most common presentation in those with well-controlled HIV, and are not suitable to monitor change over time [6–12]. At this point, clinicians and persons living with HIV are concerned about the potential development of cognitive difficulties and we are addressing this unmet clinical need.

While a full neuropsychological evaluation is required to characterize cognitive strengths and weaknesses and make a diagnosis of HAND, simpler approaches may be adequate for detecting cognitive decline. In previous work, we used a modern psychometric method, Rasch analysis, to show that cognitive ability can be quantified as a single construct in HIV, measured with tests of processing speed, attention, memory and executive function. We demonstrated that tests assessing various cognitive domains can be aligned hierarchically (from easier to more difficult) to create a ruler of “cognitive ability”, and that the same scoring algorithm can be used when administered by different raters and to individuals who differ on age, sex, and education. This yielded a short battery of tests that could be combined to assess cognition as a single construct in HIV, the Brief Cognitive Ability Measure (B-CAM) [13].

Here we ask if this measure is stable in people with HIV tested repeatedly over a time frame when no substantial change is expected (6 months), as a first step in validating its suitability to detect change following interventions or in settings where decline is expected. Cognitive test performance may improve over time simply by virtue of practice. This has been a concern in the neuropsychological literature in HIV [14]. Practice effects can mask decline or complicate the interpretation of improvement following interventions. Practice effects may not be uniform across tests, with greater practice effects reported for tests requiring complex cognitive processing or formulation of a strategy [14–17]. Furthermore, people with strong cognitive ability at baseline may improve more on the testing with practice, potentially masking underlying decline.

Statistical corrections for mean practice effects have been used to address practice effects in HIV research [14, 18]. However, these methods do not consider individual differences in the extent of practice effects. Group-Based Trajectory Analysis (GBTA) is an alternative approach to studying longitudinal change that allows for a more individualized assessment of practice effects, identifying distinct groups characterized by their starting value, with unique patterns of change over time [19]. We have previously applied this analysis to each of the 15 neuropsychological tests administered in the CHARTER study, finding very little change over time [19]. Here, we apply GBTA to provide evidence of stability over time on a briefer cognitive ability measure (B-CAM).

A second challenge for cognitive assessment is how best to combine the scores of multiple tests. This is compounded with repeated testing because practice effects may not be uniform across tests. In measures developed using Rasch analysis, differential practice effects across test items are reflected in a change in the ordering of difficulty of the items at different administrations, so-called differential item functioning (DIF). Rasch analysis provides the information required to adjust the scoring to reflect this change in difficulty, so that multiple test forms will have equivalent difficulty. The performance of a cognitive measure developed using Rasch analysis has never been tested on repeated administration. In theory, it presents an advantage in view of potential non-uniform practice effects across cognitive tasks.

Here, we combine these two modern statistical approaches, GBTA and Rasch analysis, to improve the performance of a measure of cognitive ability in HIV, the B-CAM, in detecting change over time. A repeated-measures experiment was conducted on three forms of the B-CAM over a period in which no change in cognition is expected. We then applied GBTA to identify unique trajectories of change over time and used the trajectories as indicators of practice effects.

The aims of this study were: (i) to identify whether there was an item-specific practice effect over repeated administrations of a computerized battery of cognitive tests (B-CAM) over a six-month period; and (ii) to contribute evidence towards the presence or absence of a practice effect on the global B-CAM score.

## Methods

The Research Ethics Board of the McGill University Health Centre approved the protocol and all subjects provided written informed consent. Capacity to consent was determined by a member of the health care team who knew the patient. In case of doubt, the telephone version of the MMSE was administered and those with a score of ≤ 13/22 were excluded; this cut-off was shown to correctly identify 95.5% of individuals with cognitive impairment severe enough to interfere with the ability to provide informed consent for minimal risk studies [20, 21]. During the in-person consent process, all participants had to demonstrate that they understood the study and associated risks such as by paraphrasing the information conveyed to them.

### Design

A longitudinal study was carried out between March and September 2012 at two clinics in Montreal: the University-based Chronic Viral Illness Service (CVIS) of the McGill University Health Center, and Clinique médicale l’Actuel, a large community clinic serving the HIV+ population in Montreal. A systematic sample of people with appointments on the days during the study period was drawn by approaching every third consecutive patient. Three different forms of the computerized cognitive assessment were administered over a 6-month period by three trained research assistants, either in English or French.

### Participants

We selected participants in whom no change in cognition was expected over the study period, such that an improvement in score on the cognitive test could be interpreted as a practice effect. Inclusion criteria were: > 18 years of age, able to communicate adequately in either French or English, on a stable antiretroviral regimen and with an undetectable viral load for ≥ 6 months. Exclusion criteria were: history of neurological disorder likely to affect cognition; current dependence on alcohol or street drugs (as per DSM-IV criteria); current Axis 1 psychiatric disorder or use of psychoactive medication likely to substantially interfere with cognition.

To characterize the sample, we collected information on ethnicity, living situation, HIV-related immune markers, self-reported cognitive difficulties, mood, function and health perception. In those who were invited but declined participation, we collected age, sex, education, employment and rating of level of independence in daily living. The local Research Ethics Board approved the protocol and all participants provided informed consent.

### Measures

Three sets of measures were included in this study with data obtained through questionnaires, chart review, and observed performance.

#### B-CAM

Version 1 of the B-CAM was administered. A full description of this version has been published previously [13]. The measure was developed using Rasch analysis and all test items fit the model, indicating that a legitimate total score can be derived for the construct, i.e. cognitive ability. In previous work, we established that the B-CAM showed no DIF by age, sex, education, language of administration, or rater [22].

Measures developed using Rasch analysis can be readily streamlined, removing redundant items without sacrificing fit to the model or precision of measurement. In parallel work, we acquired data needed to streamline the B-CAM in a large cohort of people with HIV across Canada (n = 856) [23]. This resulted in a reduction of the number of cognitive tasks from 28 to 6, with no effect on item fit (B-CAM Version 2). As this shorter version is more clinically feasible, in the current analysis we focus on this subset of the cognitive tasks, that we refer to as B-CAM version 2. The cognitive tasks included a computerized version of the Corsi block task (forward and backward) [24], Eriksen flanker task-incongruent reaction time [25], mini Trail Making test B (as in the Montreal Cognitive Assessment (MoCA [26]), letter fluency (F-A-S in English, P-L-S in French, 1 minute) and word list learning with recall of 8 words. The scoring range of this version is from 0 to 24, with higher values indicating better cognitive ability.

In order to minimize known sources of practice effect, a practice round was performed before complex or timed tasks and three different forms of the B-CAM included alternate versions of word recall and verbal fluency tasks. To standardize the administration and scoring, a detailed Operations Manual was produced and reviewed in a face-to-face meeting with the research assistants, the cognitive tasks and their written instructions were programmed on a web-based computer platform, and scoring was automated for most tasks (e.g. reaction time).

Other cognitive measures: To characterize the sample, we also administered the MoCA [26] and the 20-item Patient Deficit Questionnaire (PDQ) that assesses memory (retrospective and prospective), attention, organization and planning over the previous 4 weeks; the total score ranges from 0–80, with higher scores reflecting the presence of more symptoms [27].

#### Mood

Symptoms of depression and anxiety were measured by the Hospital Anxiety and Depression Scale (HADS) [28]. The depression and anxiety subscales each comprise 7 items with scores from 0–3; a cut-off score of 8 was applied to both subscales to identify clinically significant anxiety and depression [28].

#### Function and health perception

Level of independence with instrumental activities of daily living (IADL) was documented using the Older Americans Resources and Services (OARS) IADL questionnaire, which rates how autonomously a participant can use the telephone, travel, shop, prepare meals, do housework, take medications, and handle personal finances [22]. Scores range from 0 to 14, with higher values denoting more independence. Work status was queried by a single question. The Sheehan Disability Scale was used to measure, on a 10-point scale, the extent to which cognitive symptoms have disrupted work/school, social life/leisure activities and family life/home responsibilities in the past 14 days, with higher scores denoting more interference [29]. Participants also rated their perception of their general health on a scale from 0 (the worst possible health) to 100 (the best possible health).

### Data analysis

Descriptive statistics characterized the sample. People accepting and refusing study entry were compared. Doubts about capacity to consent were never raised.

Practice effect was established both at the item level (i.e. for each cognitive task) and the level of the whole B-CAM battery (global score). At the item level, practice effect was inferred by a DIF analysis. For example, if one item is significantly easier upon repeat administration than on initial testing, while the other items maintain the same level of difficulty, the relative position of this item in the hierarchy of items at time two is expected to be lower. This change in position was tested using a 2-way analysis of variance on time and class interval, where class interval defines the group according to ability.

At the level of the measure as a whole, evolution of the total score of the B-CAM was modeled using GBTA, a longitudinal statistical approach [30]. Whereas most standard statistical approaches (including hierarchical modeling and latent curve analysis) are designed to estimate the average trajectory of the population and use covariates to explain variability about this average, GBTA assumes the sample is composed of distinct groups of individuals that follow a similar evolution or trajectory over time [31, 32]. This approach allows for the possibility that there are meaningful subgroups within a population that follow distinctive trajectories over time. The number of unique trajectories is determined by theory, the distribution of the baseline values, and model fit criteria. The parameters estimated from GBTA with 3 time points are: the intercept and its standard error (SE), the linear slope and its SE, and the average posterior probability of group membership, which is considered a measure of model fit. Posterior probabilities of mean greater that 70% indicate good fit [30]. People with missing data contribute to the trajectories. The number of time points each person contributes will affect their posterior probability of group membership. In the current context, flat trajectories are expected, and improvement of greater than half a standard deviation over baseline is interpreted as a practice effect [33].

## Results

**Fig 1:**
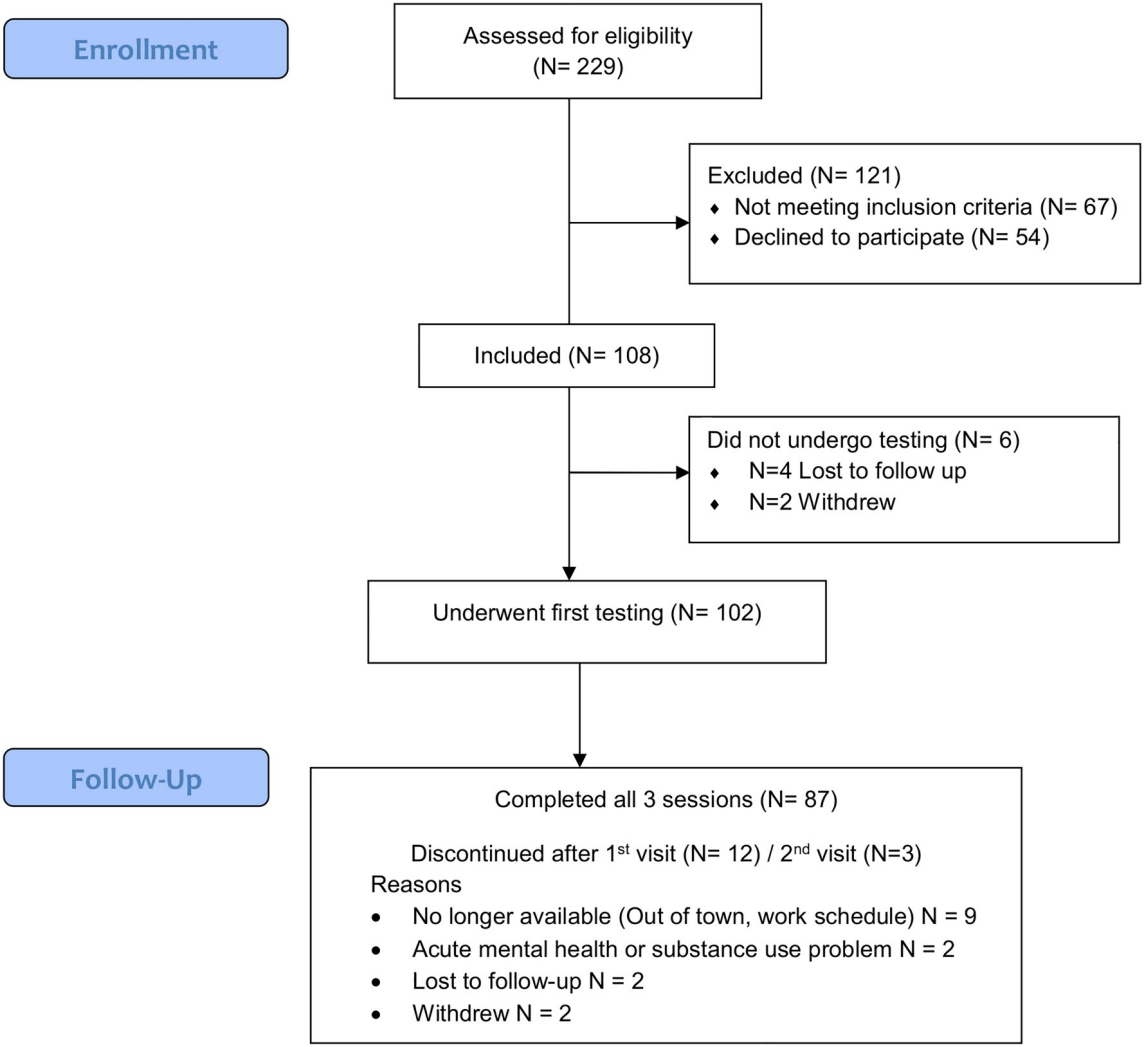
Flow diagram of recruitment.

Fig 1 shows the flow of participants during the study. Among the 229 individuals assessed for eligibility, 67 did not meet inclusion criteria, 54 declined to participate and 6 did not undergo testing. Participants and refusers had similar age (mean ± SD: 49.1 ± 9.9 y in participants, 47 ± 10.1 y in refusers, p = 0.97) and rates of employment (65% in participants, 74% in refusers, p = 0.30); however, refusers were more often women (5% in participants, 20% in refusers, p< = 0.05) and were less educated (university educated: 46% in participants; 31% in refusers; p = 0.09). Among the 102 tested participants, 15 did not complete all three study visits for reasons unrelated to the presence of cognitive difficulties, but are still included in the analysis.

Table 1 provides the socio-demographic and clinical characteristics of the sample. Most participants were French-speaking, middle-aged, educated men, who were working, living independently, reporting good general health, endorsed very few cognitive difficulties (PDQ), and had little or no limitation in the performance of everyday roles. Current CD4 cell count was in the normal range. A third of the participants had a nadir CD4 cell count < 200 cells/μL. Rates of clinically significant symptoms of depression and anxiety were high (21% and 42% respectively). In keeping with the multi-ethnic nature of the Montreal population, participants were born in 18 different countries outside North America and as many as 25% of the participants were not tested in their first language.

**Table 1:** Socio-demographic and clinical characteristics of the sample (N = 102).

Characteristic	N (%) or Mean ± SD
**Demographics**	
Sex, male	97 (95)
Age, Mean ± SD	49 ± 9.9
Age, years N (%)	
< 45	34 (33.3)
45–55	44 (43.1)
> 55	25 (24.5)
Education years, N (%)	
< 7	0
8–11	20 (19.6)
12–15	35 (34.3)
>15	47 (46.1)
Born in Canada, N (%)	75 (73.5)
Self-reported ethnicity, White N (%)	80 (78.4)
Living with	
Alone	51 (46.3)
Partner	33 (30.6)
Family members/others	18 (17.6)
Assessed in French, N (%)	78 (80.5)
**HIV Immune Markers**	
Current CD4 cell count (cells/μL), Mean ± SD	651±254
Nadir CD4 cell count (cells/μL), Mean ± SD	273 ±151
**Cognitive Measures**	
B-CAM score (0–24)	14.4 (4.4)
Montreal Cognitive Assessment^1^ (0–30), Mean ± SD	26 ± 2.2
Perceived Deficits Questionnaire^2^ (0–80), Mean ± SD	23.8 ± 13.7
**Mood**	
HADS-D Depressed^3^, (N)%	21 (19.8)
HADS-A Anxious^3^, (N)%	42 (38.8)
**Function and Health Perception**	
OARS IADL (0–14)^1^, Mean ± SD	13.9 ± 0.4
Sheehan Disability Scale (0–10 for each domain)^2^, Median (IQR)	
Disruption work/school	0 (0–3)
Disruption social life/leisure activities	0 (0–2)
Disruption family life/home responsibilities	0 (0–3)
Working > 20 hours/week, N (%)	65 (63.7)
Rating of health (1–100)^1^, Mean ± SD	82.0 ±14.0

^1^ Higher is better

^2^ Higher is worse

^3^ Based on cut-off of ≥ 8 on HADS

The steps taken to fit the data to the Rasch model are described in Table 2.

**Table 2:** Explanation of steps taken to fit the data to the Rasch model.

Threshold order	There should be a logical ordering to the values that the person achieves such that achieving a more optimal response level should situate the person at a higher level of the latent trait. For items that have a continuous scale, uniform ordinal cut-points are created that represent the distribution of the response scale. On these ordinal categories, a person with higher cognitive ability is expected to achieve a higher response value. At lower ability, the person should achieve a lower response level. If the thresholds are disordered, the response categories need to be grouped to create ordered categories, sometimes reducing the responses to binary. The number of thresholds is equal to the number of response options—1 and reflects the number of “jumps” the person has to make for each item.
Fit to the Rasch model	The items should line up hierarchically such that those items that need little ability to achieve the most optimal response level are at the low end and those items requiring more ability to achieve are higher. Overall goodness of model fit is indicated by a non-significant Chi-square test (p>0.05) after a Bonferroni adjustment for the number of items. Fit of each item and each person is as important, or even more important, than overall fit. Item and person fit is indicated when fit residual (deviance from pure linearity) values are within ±2.5 and the Chi-square test for fit is non-significant (>0.05). Those items that fail this criterion need to be looked at carefully to ensure their importance in scoring the latent trait. A fit residual of >+2.5 indicates the item does not fit the latent trait; a fit residual of <-2.5 indicates the item overfits and may be redundant.
Unidimensionality	A requirement of the Rasch model is that a single latent trait is being measured. This is assessed using a principal component analysis (PCA) of the fit residuals. The person-ability estimates derived from all pair-wise comparisons of the two most disparate set of items (those with the highest positive and negative loadings on the first factor) are compared using independent t-tests. For a set of items to be considered unidimensional, less than 5% of *t* values should be outside ± 1.96. When this value is greater than 5%, a binomial test of proportions is used to calculate the 95% confidence interval (CI) around the *t*-test estimate. Evidence of unidimensionality is still supported if the 5% value falls within the 95% CI.
Response dependency	Uniqueness of the information provided by the items is a requirement of the Rasch model. Items with pair-wise residual (after controlling for the latent trait) correlations greater than 0.3 could indicate lack of independence of the responses which inflates the reliability. Solutions include creating a super-item which combines the response options across items or choosing the one item that best suits the testing context.
Differential item functioning (DIF)	The items should have the same ordering of difficulty across all people being measured defined by personal factors such as in this study, education or age. DIF is an indicator of item bias. Typically, DIF is indicated with a significant F-test from a two-way analysis of variance. A caution is that with large and sample sizes anything may be significant; with small sample sizes, nothing may be significant. Commonly used statistical packages provide a way of visually inspecting DIF (item characteristic curves are plotted by the level of each factor will support or not the information from the statistical approach). Two options are available for items with DIF, deletion or split scoring.
Targeting	An ideally targeted measure should include a set of items that spans the full range of the theoretical latent construct (-4 to +4 logits), and have a mean location of 0 with a standard deviation (SD) of 1. Ideally, the person estimates from this measure should be centered on location 0 with a SD of 1.
Discrimination or person-separation	This indicates how well people are differentiated by the spread of the item-difficulty. The person-separation index (PSI) is interpreted like a Cronbach’s alpha. The larger the index, the better is the discrimination which facilitates the measurement of change. Values of >0.9 are suitable for measuring within-person change, values >0.7 are suitable for detecting group differences.

Practice effect was first tested at the item level. There was DIF by testing session on two items, raising the possibility of an item-specific practice effect, but this was completely confounded by test form. The fluency test used at session 2 was much easier than the test at sessions 1 and 3. Likewise, recall of the three different lists of eight words had different levels of difficulty, despite the selection of items with similar linguistic properties. These two items were split by testing session to control for DIF by form, such that a different level of difficulty was assigned to the same item for sessions 1, 2 and 3. For all other items, the hierarchy remained constant between all three time points, indicating that practice effect, if present, was homogeneous across items.

**Fig 2:**
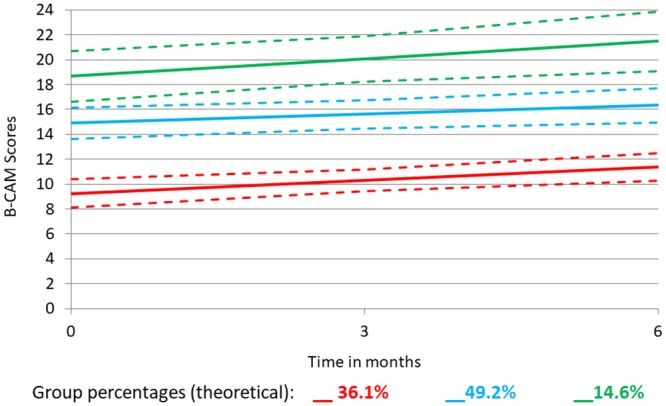
Group-based trajectory analysis of B-CAM score over 3 time points (0, 3, 6 months). Solid lines show means, dashed lines show the 95% confidence intervals. Percentage of the sample in each group (theoretical) is shown in the legend.

Practice effect on the global B-CAM score was then estimated with GBTA. As can be seen in Fig 2 and Table 3, the evolution of the global score was not uniform across groups.

**Table 3:** Evolution of the global score on the B-CAM across groups.

Group	Group membership (%)	Intercept (SE)	Slope (SE)	Change over time (SE)*
1	36.1	9.2 (0.58)	0.36 (0.12)	2.16 (0.72)
2	49.2	14.9 (0.64)	0.24 (0.11)	1.44 (0.66)
3	14.6	18.7 (1.03)	0.47 (0.21)	2.82 (1.26)

* Criterion value: 2.2 (½ SD)

The sample is composed of three distinct groups of people with unique trajectories, defined mainly by their intercept: 9; 15, and 19 out of a maximum score of 24. For ease of interpretation, the beta coefficients were multiplied by 6 (months) to reflect the change over the full time period. The group with the lowest initial score (9) comprised 36.1% of the sample and showed no change with time (β: 0.36; SE: 0.12; 0.36*6 = 2.16); a middle group comprising 49.2% of the sample also showed no change (β: 0.24; SE: 0.11; 0.24*6 = 1.44). The highest group (14.6%), showed a significant linear slope (β: 0.47; SE: 0.21), with change over 6 months estimated at 2.8 (0.47 * 6) units, higher than our a priori criterion for clinical relevance (i.e. grater than half a standard deviation, 2.2). To identify whether the changes observed in the highest trajectory group are greater than expected from measurement error, we used a Bland-Altman plot and found that no observations exceeded the 95% confidence bands, i.e. the observed change is within measurement error.

## Discussion

This study was designed to identify practice effects on a cognitive test. The B-CAM was administered three times over 6 months in a sample of people with HIV selected to be stable over this timeframe. We found no evidence of practice effect at the item level. The two items showing DIF by time were those that had different forms, by design: letter fluency and word recall. At the level of the global B-CAM score, we found no evidence of a practice effect, defined as a change greater than half a standard deviation. One group did show some change exceeding that criterion, but this remained within the range of measurement error.

The B-CAM was developed specifically to measure change over time. Hence, a practice effect would be undesirable. In order to maintain content coverage of the cognitive domain, we included some cognitive tasks with known practice effects, i.e. word fluency and recall, but used different forms over time. This required a different scoring algorithm for each form. We avoided tasks where a general strategy could be learned.

The findings here are compatible with our results applying GBTA to the neuropsychological tasks used in CHARTER, a longitudinal study of people living with HIV in the United States, where improvement in performance on repeat administration over a 3-year period was most often within measurement error [19]. Other studies of people with HIV have also found that apparent differences on repeat neuropsychological testing were within measurement error (90% confidence intervals) [34]. This error needs to be considered in the interpretation of change in raw scores.

The present study expands on our previous findings on the application of Rasch Measurement Theory to develop a brief instrument to measure cognition in people with HIV over time in the clinic setting. We previously reported that performance on several cognitive tasks can be combined using Rasch analysis to produce a single score of cognitive ability, and that the scoring of the items can be the same when administered by different raters and to individuals who differ on age, sex, and education [13]. The present study contributes two important additional findings.

First, different scoring algorithms are required for different versions of the memory and fluency tasks. Application of Rasch analysis provides precise indication of the relative difficulty of each item compared to the others. Whereas the same norms are usually applied in interpreting performance on the F, A, S letter fluency task, we found in our sample that some letters were easier than others. The same is true for memory testing: even thought the word lists were composed of words with similar linguistic characteristics, spontaneous recall of these lists showed different levels of difficulty. No DIF was identified by age, education, ethnicity or rater in the other tasks. This absence of DIF is a desirable feature as it indicates that the same measure can be used for samples that differ demographically and be administered by different raters without affecting the results.

Second, we observed a practice effect upon repeat administration for the global B-CAM score, that was not uniform across individuals: it was larger in those with a higher baseline score. Our methodology supports a quantification of this effect that is more precise than a mean practice effect: Whereas, on average, the difference between first and last testing session is non-significant in the group overall (p = 0.06, 1-tailed), GBTA identified three different magnitudes of practice effect, specific to the initial level of cognitive ability. That is, those with the initial highest cognitive ability showed the largest practice effect. This permits a more accurate correction for person-specific practice effects on repeat testing.

Our study has several strengths. The people who participated did not differ significantly from those who declined participation [35]. As such, the sample is representative of the population of HIV+ individuals for whom we are developing this test, i.e. those on a stable antiretroviral regimen, with an undetectable viral load for ≥ 6 months and no major confounding condition such as substance abuse or neurological condition that might substantially affect cognition, followed in specialty HIV clinics in Canada. Attrition was minimal and unrelated to the presence of cognitive difficulties, such that the sample at Time 3 was not different than at Time 1. Participants were from very diverse background, born in 18 different countries outside North America, and 24% were not tested in their first language. Participants also presented a range of depressive and anxiety symptoms. The B-CAM can therefore be used in patients from different cultural backgrounds and with a range of mood symptoms, with no change in scoring. Our recruitment approach resulted in the enrolment of very few women, given the demographics of the clinics’ clientele. More work is needed to assure that practice effects do not differ by sex, but this seems unlikely at face value, given the absence of other demographic effects.

In summary, we have applied two modern psychometric methods to characterize practice effects on the B-CAM, a measure of cognitive ability that combines several cognitive tasks in people living with HIV. There was DIF only for two items, related to the level of difficulty of alternate versions of these tasks. These data allow the scoring algorithm to be modified to correct for this variation in difficulty, so that all versions are strictly equivalent. GBTA showed that the three versions of the B-CAM can be administered without correction for practice effect, as the observed improvements in total scores fell within measurement error. The results provide detailed information about the expected change over time, if more precise estimates are required. Work is needed now to determine if this cognitive ability measure can detect change when change is expected. The results here are a further step towards a measure of cognitive ability able to detect meaningful change, and feasible for use in typical clinical or research settings, a necessary pre-condition for understanding, following and treating cognitive difficulties in the HIV+ population.
